# Modern Sources of Pin Money

**Published:** 1884-07

**Authors:** 


### MODERN SOURCES OF PIN MONEY.

THERE are hundreds of ladies in the city of Albany, far removed from necessity, who turn their skill, ingenuity, and energy to account in these æsthetic times, of whose handwork little is known except to themselves, but the profit on which is a very handsome penny, said a gentlemen whose relations with an enterprise in this city makes a large amount of information available to him. "I have means of informing myself regarding the extent of this thing, and have been surprised to find it so general a practice.

Ten years ago a lady who worked with her hands would be frowned down in society. Now many ladies cultivate some of the fine arts for no other purpose in the world than to replenish their purses by the employment of leisure time. Painting and embroidery are the most popular forms of art work, chiefly, perhaps, because anything good in these lines, particularly that created by the needle, finds ready sale at good round prices. The decoration of menu cards and china is also a remunerative occupation, yielding excellent pay for fine work. The art rooms of Boston, New York, Philadelphia, and our own city accept these products on commission. I understand that a young lady living in Albany wields one of the daintiest brushes in applying colors to menu cards. She disposes of all her work in New York, by means of the Decorative Art Rooms. Several ladies not only do embroidery for sale, but one or two of the deftest give lessons in the art, and get a nice return for teaching others what they know. It sounds odd, but some of the nicest people in Albany sell their handiwork. They do it occasionally as a gratification of their pride, to convince themselves that if put to it they could earn their own living. These are what are called in polite circles aristocratic arts."

"Is not the prevalence of the custom fatal to its probable continuance?"

"It is very apt to be, that is true, but within a year a new art has been developed calculated to relieve the pressure on the embroidery and painting business. High art has been introduced into the preservation of fruits and the baking of cakes and sweetmeats. This too has become a popular employment with people who are able to acquire the skill necessary. At the various art rooms you will find canned fruit and fancy bake stuffs on exhibition, made by ladies for the most part who do it to employ their leisure time. These goods command the highest prices, being bought at rates which dealers in the stores would not dare to ask. The ill-success of so many people in making their preserves makes this business supply something of a want. Cake baking, too, is an equally profitable venture. I see advertised in the Albany papers the wares of a lady resident in a city in Central New York, who supplies cakes to a vast number of customers. She made herself famous by baking what is called 'Angel's Food.' She is a member of a family who were once immensely wealthy, but are now reduced to straitened circumstances. Her business is worth $5,000 a year to her at present and is growing all the time. Another case is that of a lady who puts up canned fruits. She is the daughter of an ex-Congressman, and is married to the son of the greatest diplomat America has produced in recent years. There is no need of her applying her skill in this manner, but she was induced to make a business of what was a pastime by the urgent solicitation of friends who ate her preserves. She now gets a very handsome revenue from the work."—*Albany Journal.*

